# Diagonal ear lobe crease in diabetic south Indian population: Is it associated with Diabetic Retinopathy?. **S**ankara **N**ethralaya **D**iabetic **R**etinopathy **E**pidemiology **A**nd **M**olecular-genetics Study (SN-DREAMS, Report no. 3)

**DOI:** 10.1186/1471-2415-9-11

**Published:** 2009-09-29

**Authors:** Rajiv Raman, Padmaja Kumari Rani, Vaitheeswaran Kulothungan, Tarun Sharma

**Affiliations:** 1 Shri Bhagwan Mahavir Vitreoretinal services, 18, College Road, Sankara mx vir Vitreoretinal services, 18, College Road, Sankara Nethralaya, Chennai-600 006, Tamil Nadu, India; 2 Department of Molecular Genetics, 18, College Road, Sankara Nethralaya, Chennai-600 006, Tamil Nadu, India

## Abstract

**Background:**

To report the prevalence of ear lobe crease (ELC), a sign of coronary heart disease, in subjects (more than 40 years old) with diabetes and find its association with diabetic retinopathy.

**Methods:**

Subjects were recruited from the Sankara Nethralaya Diabetic Retinopathy Epidemiology And Molecular-genetics Study (SN-DREAMS), a cross-sectional study between 2003 and 2006; the data were analyzed for the1414 eligible subjects with diabetes. All patients' fundi were photographed using 45° four-field stereoscopic digital photography. The diagnosis of diabetic retinopathy was based on the modified Klein classification. The presence of ELC was evaluated on physical examination.

**Results:**

The prevalence of ELC, among the subjects with diabetes, was 59.7%. The ELC group were older, had longer duration of diabetes, had poor glycemic control and had a high socio-economic status compared to the group without ELC and the variables were statistically significant. There was no statistical difference in the prevalence of diabetic retinopathy in two groups. On multivariate analysis for any diabetic retinopathy, the adjusted OR for women was 0.69 (95% CI 0.51-0.93) (p = 0.014); for age >70 years, 0.49 (95% CI 0.26-0.89) (p = 0.024); for increasing duration of diabetes (per year increase), 1.11(95% CI 1.09-1.14) (p < 0.0001); and for poor glycemic control (per unit increase in glycosylated heamoglobin), 1.26 (95% CI 1.19-1.35) (p < 0.0001). For sight-threatening diabetic retinopathy, no variable was significant on multivariable analysis. In predicting any diabetic retinopathy, the presence of ELC had sensitivity of 60.4%, and specificity, 40.5%. The area under the ROC curve was 0.50 (95% CI 0.46-0.54) (p 0.02).

**Conclusion:**

The ELC was observed in nearly 60% of the urban south Indian population. However, the present study does not support the use of ELC as a screening tool for both any diabetic retinopathy and sight-threatening retinopathy.

## Background

Coronary heart disease (CHD) is a leading cause of mortality in persons with type 2 diabetes [1]. In the general population, macrovascular disease is the primary pathogenic mechanism causing CHD; however, in population with diabetes, it is the microvascular disease, in addition to the macrovascular component, that may play a role in the development of CHD. Recent evidences from the ARIC (Atherosclerosis Risk In Communities) study suggest that the presence of diabetic retinopathy (microvascular disease) poses a two-fold higher risk of CHD (macrovascular disease) and a three-fold higher risk of fatal CHD; therefore, there is a link between macrovascular and microvascular pathogenic mechanisms [2].

The presence of ear lobe crease (ELC) and its association with CHD was first described in 1973 [3]. Blodgett et al found that 75% of CHD cases had ear lobe crease as compared to 35% of the controls (age and gender matched) [4]. Hence, there is a link between ELC and macrovascular disease (CHD), and between, microvascular disease and macrovascular disease. But what link we have between ELC and microvascular disease, we do not know. The present study is aimed to find out the prevalence of ELC in south Indian diabetic population and its association with diabetic retinopathy in a population-based study.

## Methods

The study design and research methodology of SN-DREAMS 1 are described in detail elsewhere [5]. The sample was stratified based on the socio-economic scoring: low (score, 0-14), middle (score, 15-28), and high (score, 29-42) [6].

The scoring was calculated on the basis of several parameters such as ownership/type of residence (rent or own), number of rooms in the house, educational status, salary, occupation, material possessions (cycle, TV, audio, car etc.) and house/land value. Eligible patients, above 40 years, were enumerated using the multistage random sampling method. For all those who were not previously diagnosed to have diabetes diabetic, fasting blood glucose estimation was done twice: first, in the field using capillary blood, and second, at the base hospital using laboratory method (glucose oxidase method) [7]. Patients were considered to be newly diagnosed with diabetes, if the fasting blood glucose level was ≥110 mg/dl on two occasions, as described above [8]. The study was approved by the Institutional Review Board, and an informed consent was obtained from all individuals.

Demographic data, socio-economic status, physical activity, risk of sleep apnea, dietary habits, and anthropometric measurements were collected. A detailed medical and ocular history and a comprehensive eye examination, including stereo fundus photographs, were taken at the base hospital. Biochemical investigations (Blood sugar, total serum cholesterol, high-density lipoproteins, serum triglycerides, hemoglobin, glycosylated hemoglobin HbA1c) were conducted at the base hospital in fasting state.

The presence of a diagonal ear lobe crease was assigned to a person with a crease stretching obliquely from the outer ear canal towards the border of the ear lobe of both the ears; examiners were guided to diagnose the ELC, by comparing the features with the standard photograph provided to them.

Diabetic retinopathy was clinically graded using Klein's classification (Modified Early Treatment Diabetic Retinopathy Study scales) [9]. The alternative method involves grading all stereoscopic standard fields as a whole, and assigning a level of severity for the eye according to the greatest degree of retinopathy using a modified Airlie House Classification scheme. Retinal photographs were taken after pupillary dilatation (Carl Zeiss Fundus Camera, Visucamlite, Jena, Germany); all patients underwent 45° four-field stereoscopic digital photography (posterior pole, nasal, field, superior, and inferior). All photographs were graded by two independent observers in a masked fashion; the grading agreement was high (k = 0.83) [5].

Sight-threatening diabetic retinopathy was defined as the presence of severe non-proliferative diabetic retinopathy, proliferative diabetic retinopathy and clinically significant macular edema [10].

Of the 5,999 subjects enumerated, 5,778 (96.32%) responded for the first fasting blood sugar estimation. Of the 5,778 subjects, 1,816 individuals (1,349 with known history of diabetes and 467 with provisionally diagnosed diabetes) were invited to visit the base hospital. Of the 1,563 (85.60%) who responded, 138 were excluded; in two, the age criteria was not met, and in 136, the second fasting blood sugar was ≤ 110 mg/dl). Provisionally diagnosed diabetes was defined as new asymptomatic individual with a first fasting blood glucose level ≥ 110 mg/dl. An additional 11 individuals were excluded as their digital fundus photographs were of poor quality, making them ungradable for further analysis. Thus, a total of 1,414 individuals were analyzed for the study.

## Results

Of the 1,414 subjects who were analyzed for diabetic retinopathy, the mean age was 56.3 ± 10 yrs; 750 (53.04%) were men, and 664 were (46.96%), women. The prevalence of ELC in the diabetic population was 844 (59.7%) (95% CI 57.1 - 62.2)); ELC was evident in both the ears, in all subjects. Diabetic retinopathy was seen in 255 (18.03%) (95%CI 16.06-20.13) subjects.

Table 1 compares the clinical and laboratory data between the ELC group and the group without ELC. The subjects in ELC group (844) were older, had longer duration of diabetes, had poor glycemic control, and had a high socio-economic status as compared to the group without ELC (570); all variables were statistically significant. No differences were observed between the two groups with regard to gender, smoking and alcohol, diabetic retinopathy, BMI, Blood pressure, serum lipids and microalbuminuria.

**Table 1:** Clinical and Laboratory characteristics in ELC group versus non-ELC group

	**ELC Group (Present) n = 844**	**ELC Group (absent)** **n = 570**	**P value**
**Age (years)**	57.39+/-10.13	54.75+/-9.65	***<0.0001***
***Gender***			
Male	463 (54.9)	287 (50.4)	0.1076
Female	381 (45.1)	283 (49.6)	0.1076
**Smoking**	169 (20.0)	108 (18.9)	0.6576
**Alcohol**	175 (20.73)	135 (23.68)	0.2114
***Retinopathy group***			
No DR	690 (81.8)	469 (82.3)	0.8654
Any DR	154 (18.2)	101 (17.71)	0.8657
NST	124 (14.7)	91 (16.0)	0.5538
ST	30 (3.6)	10 (1.8)	0.0680
**Duration of DM (months)**	79.90+/-76.66	70.86+/-73.91	***0.0275***
**BMI (Kg/m^2^)**	25.48+/-4.16	25.22+/-3.98	0.2410
**BP Systolic (mmHg)**	139.51+/-21.13	138.57+/-20.27	0.4044
**BP Diastolic (mmHg)**	81.98+/-11.33	81.93+/-11.44	0.9354
**Glycosylated Hb**	8.34+/-2.25	7.99+/-2.11	***0.0033***
**Serum cholesterol (mg/dL)**	186.77+/-39.77	186.16+/-42.19	0.7826
**Serum HDL (mg/dL)**	39.35+/-9.66	39.06+/-10.91	0.5994
**Ratio HDL/cholestrol**	0.200+/-0.059	0.204+/-0.064	0.2272
***SES***			
Lower	653 (77.36)	500 (87.71)	***<0.0001***
Upper	191 (22.6)	70 (12.3)	***<0.0001***
***Microalbuminuria***			
Normal	681 (80.7)	469 (82.3)	0.4914
Microalbuminuria	139 (16.5)	87 (15.3)	0.6670
Clinical microbuminuria	24 (2.8)	14 (2.5)	0.8612

ELC: Ear lobe crease, DR: Diabetic retinopathy, NST: Non-Sight threatening diabetic retinopathy including mild and moderate non-proliferative diabetic retinopathy, ST: Sight threatening diabetic retinopathy including severe non-proliferative diabetic retinopathy, proliferative diabetic retinopathy and diabetic macular edema, DM: Diabetes mellitus, BMI: Body mass index, BP: Blood pressure, Hb: Heamoglobin, HDL: High density lipoprotein, SES: Socioeconomic status.

Table 2 shows the results of logistic regression analysis, keeping diabetic retinopathy as an outcome variable. For any diabetic retinopathy, the univariate analyses identified several associated factors: women OR 0.64 (95% CI 0.49-0.85) (p = 0.002), for 50-59 years, OR was 1.7 (95% CI 1.18-2.44) (p = 0.012), for 60-69 years, OR was 1.7 (95% CI 1.13-2.45) (p = 0.010), increasing duration of diabetes per year OR 1.1 (95% CI 1.09-1.13) (p < 0.0001), and poor glycemic control (per unit increase in glycosylated heamoglobin) OR 1.3 (95% CI 1.21-1.36) (p < 0.0001). In multivariate analysis, the adjustments were done for age, gender, duration of diabetes, serum lipids, blood pressure, socio-economic status and glycemic control. From the multivariate analysis, the adjusted OR for women was 0.69 (95% CI 0.51-0.93) (p = 0.014); for age >70 years, 0.49 (95% CI 0.26-0.89) (p = 0.024); for increasing duration of diabetes (per year increase), 1.11(95% CI 1.09-1.14) (p < 0.0001); for presence of ELC, 0.88 (95% CI 0.65-1.19) (p = 0.422) and for poor glycemic control (per unit increase in glycosylated heamoglobin), 1.26 (95% CI 1.19-1.35) (p < 0.0001).

**Table 2:** Regression Analysis to study the effect of various risk factors on Diabetic Retinopathy and Sight-threatening Diabetic Retinopathy.

	**Diabetic Retinopathy**						**Sight-threatening Diabetic Retinopathy**					
		
	**Univariate Analysis**			**Multivariate analysis**			**Univariate Analysis**			**Multivariate analysis**		
	
	**OR**	**95% CI**	**p**	**OR**	**95% CI**	**p**	**OR**	**95% CI**	**p**	**OR**	**95% CI**	**p**
**Gender**												
Men	1			1			1			1		
Women	0.64	0.49-0.85	**0.002**	0.69	0.51-0.93	**0.014**	0.86	0.42-1.73	0.667	0.92	0.44-1.91	0.822
												
**SES**												
Lower	1			1			1			1		
Upper	0.09	0.63-1.29	0.584	0.72	0.48-1.07	0.104	1.24	0.53-2.92	0.617	1.00	0.39-2.52	0.996
												
**Ear lobe crease**												
Absent	1			1			1			1		
Present	1.036	0.79-1.37	0.800	0.88	0.65-1.19	0.422	2.02	1.02-4.73	**0.043**	2.00	0.91-4.34	0.086
												
**Age groups (years)**												
40-49	1			1			1			1		
50-59	1.698	1.18-2.44	**0.012**	1.38	0.94-2.04	0.100	2.39	0.76-7.15	0.135	2.13	0.66-6.80	0.204
60-69	1.665	1.13-2.45	**0.010**	1.08	0.71-1.66	0.710	2.86	0.88-9.24	0.079	2.43	0.73-8.08	0.148
≥ 70	1.120	0.66-1.88	0.673	0.49	0.26-0.89	**0.024**	3.22	0.78-13.30	0.106	2.83	0.65-12.32	0.167
												
**Duration of Diabetes (per year increase)**	1.110	1.09-1.13	**<0.0001**	1.11	1.09-1.14	**<0.0001**	1.050	1.00-1.10	**0.046**	1.04	0.99-1.09	0.123
												
**HbA1c (per unit increase/**	1.280	1.21-1.36	**<0.0001**	1.26	1.19-1.35	**<0.0001**	1.060	0.93-1.22	0.383	1.07	0.93-1.25	0.343

OR: Odds Ratio, SES: Socioeconomic status, CI: Confidence interval, HbA1c: Glycosylated heamoglobin

For sight-threatening diabetic retinopathy, the univariate analysis revealed the following risk factors: presence of ear lobe crease OR was 2.02 (95% CI 1.02-4.73) (p = 0.04) and the OR for the increasing duration of diabetes (per year) was 1.05 (95% CI 1.00-1.10) (p = 0.045). No variable was significant on the multivariable analysis. The adjusted OR for ELC was 2.00 (95% CI 0.91-4.35) (p = 0.086).

Compared to the gold standard -- photographic classification -- in diagnosing sight-threatening diabetic retinopathy, the presence of ELC had a sensitivity of 75%, and specificity of 42.3%. The positive predictive value was only 19.8%. The area under the ROC curve was 0.59 (95% CI 0.50-0.68) (p = 0.047). In predicting any diabetic retinopathy, the presence of ELC had sensitivity of 60.4%, and specificity, 40.5%. The area under the ROC curve was 0.50 (95% CI 0.46-0.54) (p 0.02).

## Discussion

The diagonal ELC has been suggested as a simple marker of vascular disease in the general population, but in population with diabetes (population with increased risk of microangiopathy) only limited data are available [11]. The Fremantle diabetes study reported the prevalence of ELC to be 55% in the western Australian population [11]. Our data suggest that the ELC was present in 59.7% of the diabetic population > 40 years in the urban south Indian population.

The present study shows that the subjects in the ELC group were older, had longer duration of diabetes and had poor glycemic control. Similar observations were found in the Fremantle study [11]. Subjects with ELC had a higher socio-economic status as compared to the group without ELC; this could be an indirect measure of the population that is at a greater risk for coronary artery disease.

With regards to the association between ELC and any diabetic retinopathy, we noted that increasing age, poor glycemic control and increasing duration of diabetes were significant variables in both univariate and multivariate models. Similar observations were made in other population-based studies on diabetic retinopathy [12-15].

Regarding the association between ELC and sight-threatening diabetic retinopathy, the univariate analysis showed that subjects with ELC had almost twice the risk of developing sight-threatening diabetic retinopathy. The possible explanations for this association include loss of elastin; this might be responsible for ear lobe crease and similar loss of elastin in a retinal blood vessel might account for increased leakage and dilatation. The other speculation is the ischemia; the focal ischemia of the dermal fat might cause ear lobe crease, and ischemia in retina causes sight- threatening changes. However, these speculations need further studies. Further, in multivariate analysis, when the effect of other variables was adjusted, the association between the ELC and the sight-threatening diabetic retinopathy was not significant. Taking into consideration the low sensitivity and specificity value along with low positive predictive value, the presence of ELC cannot be used as a screening tool to predict diabetic retinopathy, including sight-threatening diabetic retinopathy.

The strength of this study was that it used photography and standard grading techniques for diabetic retinopathy. Further, the study was representative of a large population and results could be extrapolated to the whole of urban India. One of the limitations of the study was not studying the non-diabetic population with regards to ELC; it would have been interesting to compare the findings with subjects without diabetes. The second limitation was not grading ear lobe crease; recent evidence has pointed out to the relationship between the increasing grades of ELC and the increasing severity of coronary artery disease[16]. Thirdly, the sample in the subgroup of sight-threatening diabetic retinopathy is small; -thus, a lower power in this subgroup analysis.

## Conclusion

ELC is present in nearly 60% of urban south Indian population with diabetes, aged above 40 years. The presence of ELC is somewhat related to sight-threatening diabetic retinopathy on univariate analysis.

## Competing interests

The authors declare that they have no competing interests.

## Authors' contributions

RR carried out the clinical evaluation in the study and wrote the manuscript. PKR participated in the design of the study, VK performed the statistical analysis and TS conceived the study, and participated in its design and coordination. All authors read and approved the final manuscript.

## Pre-publication history

The pre-publication history for this paper can be accessed here:


